# Risk of Recurrent *Staphylococcus aureus* Prosthetic Joint Infection in Rheumatoid Arthritis Patients—A Nationwide Cohort Study

**DOI:** 10.1093/ofid/ofz451

**Published:** 2019-10-19

**Authors:** Namrata Singh, Rajeshwari Nair, Michihiko Goto, Martha L Carvour, Ryan Carnahan, Elizabeth H Field, Petar Lenert, Mary Vaughan-Sarrazin, Marin L Schweizer, Eli N Perencevich

**Affiliations:** 1 Department of Internal Medicine, University of Iowa Carver College of Medicine, Iowa City, Iowa, USA; 2 The Center for Access and Delivery Research and Evaluation (CADRE), Iowa City Veterans Affairs Healthcare System, Iowa City, Iowa, USA; 3 Department of Epidemiology, College of Public Health, University of Iowa, Iowa City, Iowa, USA

**Keywords:** recurrent prosthetic joint infection, rheumatoid arthritis, *Staphylococcus aureus*

## Abstract

**Background:**

Treatment of rheumatoid arthritis (RA) often involves immune-suppressive therapies. Concern for recurrent prosthetic joint infection (PJI) in RA patients might be high and could reduce use of joint implantation in these patients. We aimed to evaluate the risk of recurrence of PJI in RA patients compared with osteoarthritis (OA) patients by utilizing a large health care system.

**Methods:**

We conducted a retrospective cohort study of all patients admitted for a *Staphylococcus aureus* PJI who underwent debridement, antibiotics, and implant retention (DAIR) or 2-stage exchange (2SE) between 2003 and 2010 at 86 Veterans Affairs Medical Centers. Both RA patients and the comparison group of osteoarthritis (OA) patients were identified using *International Classification of Diseases*, Ninth Revision, codes. All index PJI and recurrent positive cultures for *S. aureus* during 2 years of follow-up were validated by manual chart review. A Cox proportional hazards regression model was used to compare the time to recurrent PJI for RA vs OA.

**Results:**

In our final cohort of 374 veterans who had either DAIR or 2SE surgery for their index *S. aureus* PJI, 11.2% had RA (n = 42). The majority of the cohort was male (97.3%), and 223 (59.6%) had a methicillin-susceptible *S. aureus* PJI. RA patients had a similar risk of failure compared with OA patients, after adjusting for covariates (hazard ratio, 0.81; 95% confidence interval, 0.48–1.37).

**Conclusions:**

Prior diagnosis of RA does not increase the risk of recurrent *S. aureus* PJI. Further studies are needed to evaluate the effect of different RA therapies on outcomes of episodes of PJI.

## Teaser

Recurrence of S aureus prosthetic joint infection (PJI) was not significantly associated with presence of rheumatoid arthritis (RA) when compared with osteoarthritis. This is the first study to investigate recurrence of PJI in the national veteran RA population

A successful joint replacement procedure significantly enhances a patient’s quality of life. In 2010, in the United States alone, 332 000 total hip and 719 000 total knee arthroplasties were performed. Furthermore, the projections for 2030 are for these numbers to reach 572 000 and 3.48 million for hips and knees, respectively [1]. One of the most devastating complications of total joint arthroplasty is prosthetic joint infection (PJI). Not only does it present a challenge for the patient and surgeon, the economic costs associated with a PJI are significant [2]. Patients with rheumatoid arthritis (RA) often require orthopedic surgery such as large joint arthroplasty for better quality of life. It is estimated that about 5–7% of all patients undergoing a total hip or knee arthroplasty have underlying RA [3].

Patients with inflammatory arthritis (IA) such as RA are thought to be at a particularly higher risk for PJI compared with those without an IA [4–6]. Previous studies have shown an association between RA and development of PJI after primary joint arthroplasty [7, 8]. RA patients who have had an arthroplasty have an infection rate of ~4% after the arthroplasty [9]. RA patients have also been observed to have twice the risk of hospitalization for infection and revision for PJI compared with the general population and OA patients [5, 6, 9–11]. A cohort study conducted using Danish rheumatology registers observed an increased absolute number of PJIs among OA (1226 of 120 499) patients compared with RA (63 of 3913) patients and a higher crude incidence rate among OA compared with RA patients (10.9 vs 17.3 per 1000 person-years) [12]. The risk of recurrent infection has been reported to rise to 6% after a revision surgery in RA patients [6]. One study explored the cumulative survival rates free of treatment failure after the first episode of PJI in a cohort of patients with underlying RA and compared them with the survival rates of patients without RA. However, it was a single-center study and looked at RA patients treated in the 2002–2008 period [13]. Multicenter studies that assess the association between RA and recurrence of PJI are lacking. Our objective was to estimate the risk of recurrent *Staphylococcus aureus*—a leading cause of severe and recurrent PJI—in RA compared with OA patients among the US veteran population using a large national database, given the differences in pathogenesis between the 2 conditions, immune status of these patients, and differences in their treatment protocols.

## METHODS

### Study Design and Patient Population

All data for this cohort were extracted from the VA Informatics and Computing Infrastructure (VINCI), the Corporate Data Warehouse (CDW), and the Veterans Health Administration Medical SAS (VHA MedSAS) Inpatient Main data sets from the National Patient Care Database (NPCD). The Institutional Review Board of the University of Iowa and the Research and Development Committee of the Iowa City Veterans Affairs Medical Center (VAMC) approved this study.

This retrospective cohort study included all patients 18 years and older who were admitted for *S. aureus* PJI to 86 acute care VAMCs between January 1, 2003, and December 31, 2010. Patients were initially identified as having a PJI by the presence of ≥1 *International Classification of Diseases*, Ninth Revision (ICD-9), diagnosis code for PJI (996.6, 996.60, 996.66, 996.67, 996.69) or presumptive PJI (999.3, 998.5, 998.51, 998.59, 998.6). PJI was confirmed by the presence of at least 2 cultures positive for *S. aureus* from tissue, synovial fluid, wound, and/or blood, during the time period from 15 days before PJI hospital admission to discharge. This admission episode was considered the patient’s index admission for PJI. Patients who underwent debridement, antibiotics, and implant retention (DAIR) or 2-stage exchange (2SE) of the infected hip or knee joint prosthesis after the index PJI positive culture were included in the study cohort. Patients who had retention of the whole prosthesis or exchange of mobile parts of the device were considered to have a DAIR surgery.

Patients were prospectively followed to identify the first revision surgery after the initial PJI treatment, as identified by the ICD-9 procedure or Current Procedural Terminology (CPT) code for revision surgery during the study period. Treatment failure was defined as a composite outcome including isolation of *S. aureus* from synovial fluid, joint tissue, wound at the site of surgery, or blood with or without a repeat surgery during the 2 years after the PJI revision surgery or all-cause mortality in the 90 days after revision surgery. End of follow-up period without a failure event or death >90 days after revision surgery resulted in censoring of patients. Microbiology reports for *S. aureus–*positive cultures from index and recurrent PJI episodes, as well as the type and occurrence of revision surgery, were confirmed by manual chart review in all patients included in this study cohort.

Patients with RA were identified using ≥2 occurrences of inpatient or outpatient diagnosis by ICD-9 code 714.0 or ≥1 occurrence of the ICD-9 code with presence of a DMARD prescription before revision surgery. OA patients were identified by ≥1 occurrence of inpatient or outpatient ICD-9 diagnosis code 715.90. Patients with a diagnosis code for inflammatory arthritides like ankylosing spondylitis, psoriatic arthritis, and systemic lupus erythematosus were excluded from the study. Potential confounding factors evaluated included age, sex, methicillin resistance status of *S. aureus*, site of PJI, comorbidities (Appendix), steroid use, disease-modifying antirheumatic drug (DMARD) use, other immunosuppressant medications, and the type of revision surgery done for the index PJI. A modified version of the Acute Physiology and Chronic Health Evaluation (APACHE) III scoring system was used to determine the severity of illness at admission, as described in a previous study conducted in the VA health care system population [14, 15]. Time to first PJI was defined as early if the PJI occurred within 3 months of the primary joint arthroplasty, delayed if it occurred 3 months to 2 years after, and late if it occurred >2 years after the primary joint arthroplasty [16, 17].

### Statistical Analysis

Data analyses were conducted using SAS Enterprise Guide, version 9.4, available through the VINCI secure server. The chi-square test and Fisher exact test were used to compare dichotomous and categorical variables among the groups. Effect modification was assessed by testing for interaction of covariates. The proportional hazards (PHs) assumption was evaluated by assessing the interaction of each variable with a function of time. Kaplan-Meier (K-M) curves were created to evaluate the unadjusted probability of survival free of failure between arthritis groups. Cox proportional hazards regression models were used to compare the risk of failure between the RA and OA groups. A backward selection method was used to identify important predictors of the outcome from a saturated Cox model. The arthritis group variable was retained in all models regardless of statistical significance. Year of entry in the study cohort was included as a categorical variable (2003–2006, 2007–2012) to adjust for changes in patient demographics or treatment protocols over time. Variables identified as important predictors from the backward selection approach were included in the risk adjustment model, and adjusted hazard ratios (HRs) with 95% confidence intervals (CIs) were reported from this model.

## RESULTS

Of the 374 *S. aureus* PJI cases, 42 were categorized as RA patients (11.2%) and 332 as OA patients (88.8%). The median age at time of diagnosis of PJI (range) was 64 (40–92) years. The majority of patients in the cohort were male (97.3%), and 223 (59.6%) had a methicillin-susceptible *S. aureus* (MSSA) PJI. A greater proportion of the cohort had a knee PJI (297, 79.4%) compared with hip PJI (77, 20.6%). DAIR was the preferred surgery compared with 2SE in this cohort of patients with *S. aureus* PJI (77.5% vs 22.5%). At least 53.0% of the cohort (n = 197) was identified as having an early PJI, whereas delayed and late PJI were seen in 27.0% and 21.0% of patients, respectively. The median score for the APACHEIII was 28 with an interquartile range (range) of 20–40 (3–82), suggesting that there was variability in the severity of illness in patients at the time of admission for their revision surgery. For specific comorbidities, 323 (86.4%), 151 (40.4%), and 49 (13.1%) patients were identified as having a prior or current diagnosis of hypertension, diabetes, and renal disease, respectively. Thirteen (3.5%) patients were treated with biologics, and 34 (9.1%) patients were treated with nonbiologics before their revision surgery for the PJI episode. One hundred ninety-four (51.9%) patients were treated with steroids before the revision surgery.

RA patients were more likely to be female (7.1% vs 2.1%), to have >2 comorbidities (16.7% vs 8.7%), to have had a steroid treatment (100.0% vs 50.9%), and to be treated with a biologic (23.8% vs 0.9%) or nonbiologic (57.1% vs 3.1%) before the revision surgery compared with OA patients. Patients with RA were more likely to be in the median range of APACHEIII scores for severity of PJI at the time of admission compared with OA patients (52.4% vs 28.6%). RA and OA patients were similar in the type of revision surgery used for management of their PJI (Table 1).

**Table 1.** Patient Characteristics for Arthritis Groups

Characteristics	RA (n = 42), No. (%)	OA (n = 332), No. (%)	*P* Value
Age, y			.56
<55	5 (11.9)	55 (16.6)	
55–64	15 (35.7)	130 (39.2)	
≥65	22 (52.4)	147 (44.3)	
Female	3 (7.1)	7 (2.1)	.09
*S. aureus* type			.24
MRSA	12 (28.6)	139 (41.9)	
MSSA	30 (71.4)	193 (58.1)	
Site			.14
Hip	5 (11.9)	72 (21.7)	
Knee	37 (88.1)	260 (78.3)	
Severity of illness by APACHEIII score			.007
<28	13 (30.9)	167 (50.3)	
28–44	22 (52.4)	95 (28.6)	
≥44	7 (16.7)	70 (21.1)	
Charlson comorbidity score (>2)	7 (16.7)	29 (8.7)	.10
Hypertension	37 (88.1)	286 (86.1)	.73
Diabetes	13 (30.9)	138 (41.6)	.19
Renal disease	5 (11.9)	44 (13.3)	.81
Steroid use	42 (100.0)	169 (50.9)	<.0001
Surgery			.87
DAIR	33 (78.6)	257 (77.4)	
2-stage exchange	9 (21.4)	75 (22.6)	
Time to first PJI			.55
Early	19 (45.2)	178 (53.6)	
Delayed	14 (33.3)	88 (26.5)	
Late	9 (21.4)	66 (19.9)	
Prior biologic DMARDs	10 (23.8)	3 (0.9)	<.0001
Prior nonbiologic DMARDs	24 (57.1)	10 (3.1)	<.0001

Abbreviations: DAIR, debridement, antibiotics, and implant retention; DMARDs, disease-modifying antirheumatic drugs; MRSA, methicillin-resistant *Staphylococcus aureus*; MSSA, methicillin-susceptible *Staphylococcus aureus*; OA, osteoarthritis; RA, rheumatoid arthritis.

**Figure 1.**
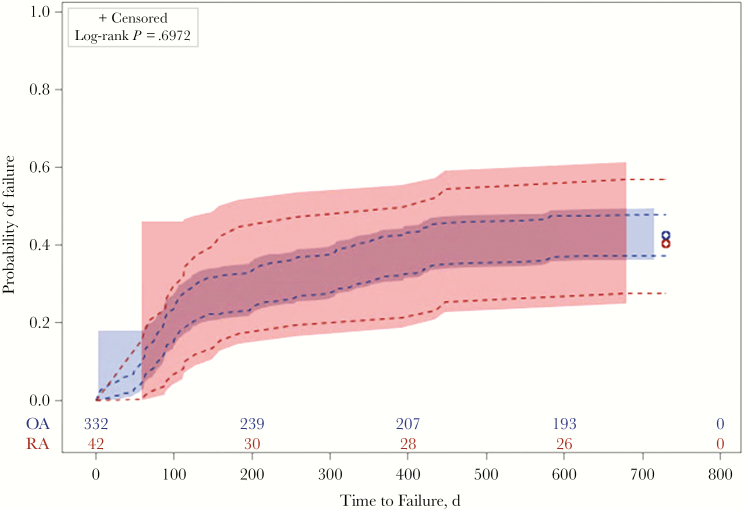
Failure curve for time to treatment failure in *Staphylococcus aureus* prosthetic joint infection patients.

**Figure 2.**
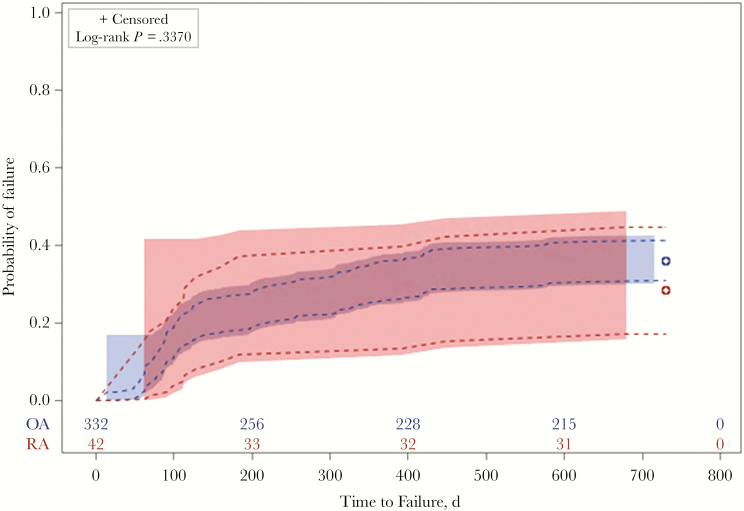
Failure curve for time to microbiological recurrence with or without revision surgery in *Staphylococcus aureus* prosthetic joint infection patients.

At the end of 2 years of follow-up, there were 158 (42.3%) treatment failures in the study cohort, of which 131 (82.9%) were failures due to microbiological relapse with or without a revision surgery. Twenty-seven of the 158 failures were due to death. RA and OA patients were not significantly different in proportion of failures during the follow-up period (40.5% vs 42.5%; *P* = .81) or proportion of failures due to microbiological recurrence of PJI (28.6% vs 35.8%; *P* = .35). The overall treatment failure rate at 2 years was 42% (95% CI, 37%–47%). Time to treatment failure (log-rank *P* = .697) and time to failure due to microbiological recurrence (log-rank *P* = .337) did not differ between RA and OA patients (Figures 1 and 2).

RA patients tended to have a lower risk of failure compared with OA patients, but this association was not statistically significant (adjusted HR [aHR], 0.81; 95% CI, 0.48–1.37) (Table 2). In the multivariate model, MRSA PJI was observed to have 59% higher risk of treatment failure compared with MSSA PJI (aHR, 1.59; 95% CI, 1.15–2.19), and DAIR was associated with a 4-times-higher risk of treatment failure compared with 2SE for the PJI (aHR, 4.42; 95% CI, 2.58–7.57). Patients with delayed and late presentation of PJI were 2 times and 2.5 times more likely to have a treatment failure compared with early PJI presentation (aHR, 1.79; 95% CI, 1.22–2.64; aHR, 2.27; 95% CI, 1.46–3.53). Diabetics were observed to have 37% higher risk of treatment failure compared with nondiabetics; however, this association was not statistically significant (aHR, 1.37; 95% CI, 0.95–1.97). Patients presenting with PJI on or after 2007 had a 32% lower likelihood of treatment failure compared with patients presenting between 2003 and 2006 (aHR, 0.68; 95% CI, 0.49–0.94).

**Table 2.** Multivariate Adjusted Cox Proportional Hazards Models for Risk of Failure in *Staphylococcus aureus* Prosthetic Joint Infection

	Crude HR (95% CI)	Adjusted HR (95% CI)
RA vs OA	0.91 (0.55–1.49)	0.81 (0.48–1.37)
Age categories, y		
≥65	0.82 (0.54–1.24)	0.90 (0.56–1.44)
55–64	0.58 (0.37–0.90)	0.60 (0.38–0.95)
<55	Reference	Reference
Methicillin susceptibility of *S. aureus*		
MRSA	1.55 (1.14–2.12)	1.59 (1.15–2.19)
MSSA	Reference	Reference
Site of PJI		
Knee	1.14 (0.76–1.71)	-
Hip	Reference	-
APACHEIII score		
≥44	1.40 (0.95–2.07)	1.07 (0.66–1.73)
28–44	1.02 (0.70–1.47)	0.93 (0.62–1.39)
<28	Reference	Reference
Charlson comorbidity score		
>2	1.40 (0.88–2.24)	-
≤2	Reference	-
Diabetes		
Yes	1.44 (1.05–1.97)	1.37 (0.95–1.97)
No	Reference	-
Steroid use		
Yes	1.28 (0.93–1.75)	-
No	Reference	-
PJI revision surgery		
DAIR	2.96 (1.79–4.89)	4.42 (2.58–7.57)
2-stage exchange	Reference	Reference
Time to PJI		
Delayed	1.19 (0.83–1.73)	1.79 (1.22–2.64)
Late	1.32 (0.89–1.96)	2.27 (1.46–3.53)
Early	Reference	Reference
Steroid use		
Yes	0.40 (0.12–1.23)	-
No	Reference	-
Prior biologic DMARD use		
Yes	0.45 (0.14–1.40)	-
No	Reference	-
Prior nonbiologic DMARD use		
Yes	0.67 (0.36–1.23)	-
No	Reference	-
Year of entry in cohort		
2007–2012	0.79 (0.58–1.09)	0.68 (0.49–0.94)
2003–2006	Reference	Reference

Abbreviations: CI, confidence interval; DAIR, debridement, antibiotics, and implant retention; DMARDs, disease-modifying antirheumatic drugs; HR, hazard ratio; MRSA, methicillin-resistant *Staphylococcus aureus*; MSSA, methicillin-susceptible *Staphylococcus aureus*; OA, osteoarthritis; RA, rheumatoid arthritis.

## DISCUSSION

Despite surgical intervention and prolonged antibiotics, 35% of the patients with an incident *S. aureus* PJI developed recurrent infection over the following 2 years. Overall, the risk of treatment failure did not statistically differ between RA and OA patients in the risk-adjusted models. These results could reflect vigilant preoperative evaluation and perioperative monitoring of RA patients, particularly in hospitals with orthopedic surgeons who have extensive experience treating RA patients with PJI. Alternatively, these results could suggest limited impact of underlying inflammatory characteristics and immunomodulatory agents used to treat RA on the risk of treatment failure or recurrence of infection after the first episode of PJI.

 PJI can be treated by different medical and surgical modalities, but DAIR and 2SE are the most common strategies. DAIR involves opening the prior incision site, thorough irrigation and debridement of any necrotic or infected soft tissue, and evacuation of any purulence surrounding the prosthesis. The joint is then closed, followed by prolonged antimicrobial therapy [2]. Patients most appropriate for this procedure are those in whom the duration of symptoms is short or those who have a stable implant and no sinus tract [2]. A 2SE involves at least 2 surgeries. First, the infected prosthesis is removed and an antibiotic-impregnated spacer is placed, followed by at least 4–6 weeks of pathogen-directed antibiotic therapy. A 2–6-week antibiotic-free period follows, during which assessment is made for ongoing signs of infection. If there are no signs of an ongoing infection, a new prosthesis is implanted. Patients eligible for this surgery are those with a sinus tract, who are ineligible for DAIR, and who are able to undergo delayed reimplantation. The treatment strategy for an individual patient is selected based on the orthopedic surgeon, patient-related factors, and with input from infectious disease physicians.

The risk of treatment failure was higher among patients managed with DAIR, regardless of the arthritis groups. Comparable results were noted in studies that did and did not include patients with inflammatory arthritis [18–21]. DAIR should be considered a surgery with risk of failure in RA patients due to persistence of resistant bacterial biofilm in patients with deficient host defenses [22–25]. Berbari et al. conducted a retrospective analysis of all patients with RA after a total hip or total knee arthroplasty infection between 1969 and 1995. This study observed lower rates of 5-year survival free of treatment failure among PJI episodes treated with DAIR (32%; 95% CI, 21%–49%) compared with those managed with 2SE (79%; 95% CI, 66%–93%) [26]. A study conducted by Hsieh et al. in Taiwan reported that RA patients had worse outcomes than non-RA patients after occurrence of a PJI [19]. This conclusion was based on patient data from a single center and was interpreted from unadjusted analyses. There are several factors that could account for differences in study findings between our study and the Hsieh study. For example, we included only index PJI episodes caused by *S. aureus* and utilized a homogenous control group of OA patients, as opposed to the Hsieh study, which had a heterogeneous control group with other inflammatory arthritides.

We observed higher risk of failure in patients with MRSA compared with MSSA in the risk-adjusted analysis. Poor penetration of anti-MRSA antibiotics, such as vancomycin, through the bacterial biofilm, along with potential for incomplete clearance of the bacteria, may result in worse outcomes among MRSA PJI patients. It is important to note that among our study patients, the majority had an MSSA PJI, suggesting that even a susceptible pathogen could place significant infection burden on an immunocompromised population. The risk of treatment failure was lower in patients treated in 2007 and later compared with those treated between 2003 and 2006. It is plausible that this reduction in treatment failure was influenced by the VHA’s bundled interventions such as universal nasal surveillance for MRSA, contact precautions for patients colonized or infected with MRSA, hand hygiene, and a change in infection control practice and outcome responsibility culture introduced in 2007 to reduce MRSA infections [27]. It is also worth noting that DAIR was the preferred surgery in our study cohort. It is plausible that patients who did not meet the criteria were also treated with DAIR due to patient or physician preference for a less invasive surgery to treat the PJI. Assessment of patients’ signs and symptoms present on admission for the revision surgery was beyond the scope of our study. DAIR was observed to be associated with increased risk of infection in our study and other published studies. Moreover, our study was conducted before the publication of the Infectious Diseases Society of America treatment guidelines for PJI. Selection of surgery type and potential nonadherence to treatment guidelines could be a few of the reasons for a high recurrence rate of PJI in our study.

To the best of our knowledge, this is the largest study to examine clinical outcomes after a PJI comparing RA with OA patients in the United States. Another major strength of our study is that the index culture result, occurrence and type of revision surgery, and clinical outcome including second positive culture for *S. aureus* during 2 years of follow-up were validated by manual chart review for all patients in the study cohort. Our study has several limitations. First, we used ICD-9 codes for identification of RA diagnosis, and there is potential for misclassification bias [28]. But specificity of the RA code in a specific population of patients undergoing arthroplasty has not been studied. Misclassification of OA patients as RA would cause findings to bias toward the null, but we tended to see better outcomes in RA patients compared with OA patients, although the results were not statistically significant. Second, the number of RA patients on DMARDs was low, so we were not able to assess the impact of an individual DMARD as a risk factor for failure. Further studies are needed that can evaluate the effect of various RA therapies on the outcomes of episodes of PJI in these patients compared with the control group. Third, our study was underpowered to identify statistically significant risk differences between RA and OA patients even though the risk estimates appeared clinically significant. Fourth, there was potential for unmeasured confounders such as obesity and smoking that could have contributed to differences between RA and OA patients in this study. Fifth, there was potential for loss of follow-up of patients who moved to a non-VA hospital for care of their PJI after the revision surgery. The findings from our study should be validated in future studies with large numbers of RA patients. Sixth, our study focused only on *S. aureus* PJI and clinical outcomes associated with *S. aureus*. However, it is worth noting that *S. aureus* is the most common cause of PJI (20%–25% cases) and has the capacity to thrive on orthopedic implants through the formation of biofilms [2]. These factors could potentially impact clinical outcomes in patients who have a deviant immune system such as in RA. Finally, the small number of female patients among US veterans and the possible higher comorbidity inherent in our cohort limit the generalizability of our findings to nonveteran populations.

In a multiyear and large multicenter cohort study, prior diagnosis of RA did not appear to increase the risk of recurrent *S. aureus* PJI. Patients with 2SE had better survival free of failure compared with DAIR regardless of the arthritis type. These findings are somewhat reassuring, as risk of recurrent PJI does not appear to be a reason to avoid prosthetic joint implantation in RA patients.

## APPENDIX. ICD-9 CODES USED FOR IDENTIFYING COMORBIDITIES

**Table:**

Diagnosis	ICD-9 Code
Hypertension	401.1, 401.9, 402.10, 402.90, 404.10, 404.90, 405.1, 405.9
Diabetes	250.0–250.3, 250.4–250.7, 250.9
Renal disease	403.11, 403.91, 404.12, 404.92, 585.x, 586.x, V42.0, V45.1, V56.0, V56.8
Myocardial infarction	410.x, 412.x
Congestive heart failure	428.x
Peripheral vascular disease	443.9, 441.x, 785.4, V43.4 Procedure 38.48
Cerebrovascular disease	430.x–438.x
Dementia	290.x
Chronic obstructive pulmonary disease	490.x–505.x, 506.4
Peptic ulcer disease	531.x–534.x
Mild liver disease	571.2, 571.4–571.6
Hemiplegia or paraplegia	342.0, 342.1, 342.9– 344.x
Malignancy	140.x–172.x, 174.x.–195.8, 200.x–208.x
Moderate liver disease	456.0–456.21, 572.2–572.8
Metastatic carcinoma	196.x–199.1
HIV/AIDS	042.x–044.x

## References

[1] Kurtz S, Ong K, Lau E, et al Projections of primary and revision hip and knee arthroplasty in the United States from 2005 to 2030. J Bone Joint Surg Am 2007; 89:780–5. 17403800 10.2106/JBJS.F.00222

[2] Tande AJ, Patel R Prosthetic joint infection. Clin Microbiol Rev 2014; 27:302–45. 24696437 10.1128/CMR.00111-13 PMC3993098

[3] Berbari EF, Hanssen AD, Duffy MC, et al. Risk factors for prosthetic joint infection: case-control study. Clin Infect Dis 1998; 27:1247–54. 9827278 10.1086/514991

[4] Morrison TA, Figgie M, Miller AO, Goodman SM Periprosthetic joint infection in patients with inflammatory joint disease: a review of risk factors and current approaches to diagnosis and management. HSS J 2013; 9:183–94. 24426866 10.1007/s11420-013-9338-8 PMC3757491

[5] Doran MF, Crowson CS, Pond GR, et al Frequency of infection in patients with rheumatoid arthritis compared with controls: a population-based study. Arthritis Rheum 2002; 46:2287–93. 12355475 10.1002/art.10524

[6] Schrama JC, Espehaug B, Hallan G, et al. Risk of revision for infection in primary total hip and knee arthroplasty in patients with rheumatoid arthritis compared with osteoarthritis: a prospective, population-based study on 108,786 hip and knee joint arthroplasties from the Norwegian Arthroplasty Register. Arthritis Care Res (Hoboken) 2010; 62:473–9. 20391501 10.1002/acr.20036

[7] Bongartz T, Halligan CS, Osmon DR, et al. Incidence and risk factors of prosthetic joint infection after total hip or knee replacement in patients with rheumatoid arthritis. Arthritis Rheum 2008; 59:1713–20. 19035425 10.1002/art.24060 PMC3923416

[8] Poss R, Thornhill TS, Ewald FC, et al Factors influencing the incidence and outcome of infection following total joint arthroplasty. Clin Orthop Relat Res 1984; 117–26. 6692605

[9] Jämsen E, Varonen M, Huhtala H, et al. Incidence of prosthetic joint infections after primary knee arthroplasty. J Arthroplasty 2010; 25:87–92. 10.1016/j.arth.2008.10.013 19056210

[10] Jamsen E, Huhtala H, Puolakka T, Moilanen T Risk factors for infection after knee arthroplasty. A register-based analysis of 43,149 cases. J Bone Joint Surg Am 2009; 91:38–47. 10.2106/JBJS.G.01686 19122077

[11] Ravi B, Escott B, Shah PS, et al. A systematic review and meta-analysis comparing complications following total joint arthroplasty for rheumatoid arthritis versus for osteoarthritis. Arthritis Rheum 2012; 64:3839–49. 23192790 10.1002/art.37690

[12] Cordtz RL, Zobbe K, Hojgaard P, et al Predictors of revision, prosthetic joint infection and mortality following total hip or total knee arthroplasty in patients with rheumatoid arthritis: a nationwide cohort study using Danish healthcare registers. Ann Rheum Dis 2018; 77:281–8. 29097373 10.1136/annrheumdis-2017-212339

[13] Hsieh PH, Huang KC, Shih HN Prosthetic joint infection in patients with rheumatoid arthritis: an outcome analysis compared with controls. PLoS One 2013; 8:e71666. 23990969 10.1371/journal.pone.0071666 PMC3753295

[14] Albertson J, McDanel JS, Carnahan R, et al. Determination of risk factors for recurrent methicillin-resistant *Staphylococcus aureus* bacteremia in a Veterans Affairs healthcare system population. Infect Control Hosp Epidemiol 2015; 36:543–9. 25682861 10.1017/ice.2015.25

[15] Knaus WA, Wagner DP, Draper EA, et al. The APACHE III prognostic system. Risk prediction of hospital mortality for critically ill hospitalized adults. Chest 1991; 100:1619–36. 1959406 10.1378/chest.100.6.1619

[16] Senneville E, Joulie D, Legout L, et al. Outcome and predictors of treatment failure in total hip/knee prosthetic joint infections due to *Staphylococcus aureus*. Clin Infect Dis 2011; 53:334–40. 21810745 10.1093/cid/cir402 PMC3148259

[17] Zimmerli W, Trampuz A, Ochsner PE Prosthetic-joint infections. N Engl J Med 2004; 351:1645–54. 15483283 10.1056/NEJMra040181

[18] Lora-Tamayo J, Senneville É, Ribera A, et al; Group of Investigators for Streptococcal Prosthetic Joint Infection The not-so-good prognosis of streptococcal periprosthetic joint infection managed by implant retention: the results of a large multicenter study. Clin Infect Dis 2017; 64:1742–52. 28369296 10.1093/cid/cix227

[19] Hsieh PH, Lee MS, Hsu KY, et al. Gram-negative prosthetic joint infections: risk factors and outcome of treatment. Clin Infect Dis 2009; 49:1036–43. 19691430 10.1086/605593

[20] Azzam KA, Seeley M, Ghanem E, et al. Irrigation and debridement in the management of prosthetic joint infection: traditional indications revisited. J Arthroplasty 2010; 25:1022–7. 20378306 10.1016/j.arth.2010.01.104

[21] Kuiper JW, Vos SJ, Saouti R, et al Prosthetic joint-associated infections treated with DAIR (debridement, antibiotics, irrigation, and retention): analysis of risk factors and local antibiotic carriers in 91 patients. Acta Orthop 2013; 84:380–6. 23848215 10.3109/17453674.2013.823589 PMC3768038

[22] Goodman SM, Figgie MA Arthroplasty in patients with established rheumatoid arthritis (RA): mitigating risks and optimizing outcomes. Best Pract Res Clin Rheumatol 2015; 29:628–42. 26697771 10.1016/j.berh.2015.09.004

[23] Goodman SM, Menon I, Christos PJ, et al. Management of perioperative tumour necrosis factor α inhibitors in rheumatoid arthritis patients undergoing arthroplasty: a systematic review and meta-analysis. Rheumatology (Oxford) 2016; 55:573–82. 26447162 10.1093/rheumatology/kev364 PMC5009446

[24] McConoughey SJ, Howlin R, Granger JF, et al. Biofilms in periprosthetic orthopedic infections. Future Microbiol 2014; 9:987–1007. 25302955 10.2217/fmb.14.64 PMC4407677

[25] Zimmerli W, Moser C Pathogenesis and treatment concepts of orthopaedic biofilm infections. FEMS Immunol Med Microbiol 2012; 65:158–68. 22309166 10.1111/j.1574-695X.2012.00938.x

[26] Berbari EF, Osmon DR, Duffy MC, et al. Outcome of prosthetic joint infection in patients with rheumatoid arthritis: the impact of medical and surgical therapy in 200 episodes. Clin Infect Dis 2006; 42:216–23. 16355332 10.1086/498507

[27] Jain R, Kralovic SM, Evans ME, et al. Veterans Affairs initiative to prevent methicillin-resistant *Staphylococcus aureus* infections. N Engl J Med 2011; 364:1419–30. 21488764 10.1056/NEJMoa1007474

[28] Singh JA, Holmgren AR, Noorbaloochi S Accuracy of Veterans Administration databases for a diagnosis of rheumatoid arthritis. Arthritis Rheum 2004; 51:952–7. 15593102 10.1002/art.20827

